# Outcomes of community-based and home-based pulmonary rehabilitation for pneumoconiosis patients: a retrospective study

**DOI:** 10.1186/s12890-018-0692-7

**Published:** 2018-08-09

**Authors:** Eric W. Tsang, Henry Kwok, Aidan K. Y. Chan, Kah Lin Choo, Kin Sang Chan, Kam Shing Lau, Chetwyn C. H. Chan

**Affiliations:** 1 The Laboratory of Neuroscience for Education, Faculty of Education, The University of Hong Kong, Hong Kong, China; 2 Department of Rehabilitation Sciences, The Hong Kong Polytechnic University, Hung Hom, Kowloon, Hong Kong, China; 3 Department of Life Science, Imperial College of London, London, UK; 4 Department of Medicine, North District Hospital, Hong Kong, China; 5 Department of Medicine, Haven of Hope Hospital, Hong Kong, China; 6 Department of Medicine, Ruttonjee Hospital, Hong Kong, China

**Keywords:** Pneumoconiosis, Physical exercise, Mental health, Health-related quality of life, Community-based pulmonary rehabilitation, Home-based pulmonary rehabilitation

## Abstract

**Background:**

Pneumoconiosis patients receive community-based or home-based pulmonary rehabilitation (PR) for symptom management and enhancement of physical and mental well-being. This study aimed to review the clinical benefits of community-based rehabilitation programmes (CBRP) and home-based rehabilitation programmes (HBRP) for PR of pneumoconiosis patients.

**Methods:**

Archival data of pneumoconiosis patients who participated in CBRP and HBRP between 2008 and 2011 was analysed. There were 155 and 26 patients in the CBRP and HBRP respectively. The outcome measures used in the pre- and post-tests were Knowledge, Health Survey Short Form-12 (SF-12), Hospital Anxiety and Depression Scale (HADS), 6-Min Walk Test (6MWT), and Chronic Respiratory Questionnaire (CRQ). Paired t-tests and the Analysis of Covariance (ANCOVA) using the patients’ baseline lung functions as the covariates were performed to examine the changes in the outcomes after completing the programmes. Hierarchical multiple regression analyses were used to examine the relationships between patient’s programme participation factors and different scores of the outcome measures.

**Results:**

After controlling for patients’ baseline lung capacities, significant improvements were revealed among patients participated in CBRP in the scores of the 6MWT, Knowledge, HADS, SF-12 PCS, and CRQ emotion and mastery. The different scores in the Knowledge and HADS were correlated with the patients’ levels of programme participation. In contrast, significant improvements were only found in the scores of the Knowledge and 6MWT among patients who participated in HBRP. The gain scores of the 6MWT were correlated with the patients’ levels of programme participation.

**Conclusions:**

Both CBRP and HBRP benefited patients’ levels of exercise tolerance and knowledge about the disease. CBRP provided greater benefits to patients’ mental and psychosocial needs. In contrast, HBRP was found to improve patients’ physical function, but did not have significant impacts on patients’ mental health and health-related quality of life. The attendance of patients and the participation of their relatives in treatment sessions were important factors in enhancing the positive effects of CBRP and HBRP. These positive outcomes confirm the value of pulmonary rehabilitation programmes for community-dwelling pneumoconiosis patients.

**Electronic supplementary material:**

The online version of this article (10.1186/s12890-018-0692-7) contains supplementary material, which is available to authorized users.

## Background

Pneumoconiosis is an occupational disease of the lungs caused by inhaling organic or non-organic dust retained in the lungs [1, 2]. Patients with pneumoconiosis typically suffer from reduced lung functions [3–5], different mood and respiratory symptoms [6], and decreased tolerance for physical exercise [5]. Together, they contribute towards the deterioration of health-related quality of life (HRQOL) [4, 7]. Patients with chronic obstructive pulmonary diseases (COPD), including pneumoconiosis, are frequently referred to pulmonary rehabilitation (PR) programmes [8]. These programmes aim to relieve symptoms and to improve capacity for exercise, emotional function, sense of control, and HRQOL [8]. The content and settings of different PR programmes, their model of delivery, and personnel involved in the delivery may vary according to local health care systems and resources [9, 10]. Most PR programmes for COPD patients include either low or high-intensity exercise training, endurance-training and strength training [9–11]. Apart from rehabilitating the physical aspect of patients, these programmes may include health education, psychosocial support, and/or nutritional counseling [10, 11]. The common settings in which PR programmes are delivered include hospital-based [12], community-based [4, 13], or patient’s home [14–18]. Different settings cater for the different needs of the patients. The common duration of PR programme is 8 weeks [19]. A recent meta-analysis reviewed 65 studies on PR programmes. It was reported that PR programmes led to significant benefits in relieving dyspnoea and fatigue, and on improving exercise capacity and HRQOL, among COPD patients [8].

In Hong Kong, PR services for patients with pneumoconiosis were funded by the Pneumoconiosis Compensation Fund Board. It is a statutory body established by the local government. Three public hospitals and 2 nongovernmental organisations (NGOs) were responsible for conducting PR services [1, 20]. PR services included two core programmes: Community-Based Rehabilitation Programme (CBRP) and Home-Based Rehabilitation Programme (HBRP).

CBRP was the standard programme consisting of protocol-based classes delivered by healthcare professionals at community centres. Typical classes include breathing re-training, exercise re-conditioning, health education, teaching energy conservation techniques and panic control skills (see Additional file 1). The duration was four to 6 weeks with a frequency of twice per week.

HBRP was designed to cater for patients unable to access community-based services due to profound incapacities. The content of the programme was customised according to the needs of the patients during home visits. Unlike the typical PR services offered by CBRP, HBRP provided additional psychological support to the patients and their family. Examples of tailored services included home modification, carer-training, and living skills-training (see Additional file 1). HBRP was delivered by healthcare professionals offering eight home visits, each lasting at least 1 hour. CBRP and HBRP were complemented by adjunctive programmes that helped pneumoconiosis patients better manage their illnesses. Detailed description of each of the programmes as aforementioned can be found in the Additional file 1.

Several systematic reviews have reported positive effects of PR programmes on patients with COPD. They include minimizing COPD symptoms, improving exercise capacities, as well as improving health-related quality of life [8, 21–25]. Previous studies as well as expert opinions have consistently shown that PR programmes did not bring about improvement in the lung functions of patients with COPD [23–26]. Nevertheless, lung function had been identified as an important factor influencing HRQOL in pneumoconiosis patients [4]. It is worthwhile to explore whether the physical and psychological benefits brought about by the PR [8, 21, 22] are independent of the patients’ initial lung functions. Ascertaining this is essential for a greater understanding of the precise benefits of PR programmes for patients with pneumoconiosis.

Moreover, the majority of previous studies had not recruited pneumoconiosis patients. This calls for an investigation on the effect of PR programmes on patients who suffered from pneumoconiosis. This study aims to examine the outcomes of both the CBRP and HBRP for pneumoconiosis patients based on archived data from 2008 to 2011 by the Hong Kong Hospital Authority. Moreover, we performed covariance analyses to examine the outcomes of CBRP and HBRP independent from patients’ baseline lung functions. The relationships among patients’ characteristics, types of program participations, and clinical outcomes were examined. The findings will pave the way for contents of future PR programmes to be enhanced for pneumoconiosis patients.

## Methods

### Subjects

From 2008 to 2011, 685 pneumoconiosis patients enrolled in the CBRP or HBRP programmes offered by three hospitals in Hong Kong. The outcomes of the programmes were captured by a voluntary assessment scheme. It covered the physical and psychosocial functions of the patients before and shortly after the rehabilitation programmes. A review of the database identified 181 patients who had complete records of all physical, mental, and HRQOL outcome measures (Table 1). These cases included 155 patients from the CBRP and 26 from the HBRP. On average, patients who completed the HBRP were older. They had lower baseline forced-expiratory volume (FEV_1_) values and higher percentages of degree of impairment (%DOIs), relative to the patients who completed the CBRP. Due to the retrospective nature of the study, ethics approval was granted on the basis of not requiring the consent from the patients for participating in the study from the Institutional Review Boards of each of the three hospitals.

**Table 1:** Demographic characteristics of the 181 pneumoconiosis patients

Type of Program	CBRP	HBRP
Number of cases	155	26
Gender (male/female)	153/2	26/ 0
Age (SD) in years	70.74 (8)	74.54 (8.3)
Baseline FEV_1_ (SD) in L/min	1.64 (0.58)	1.17 (0.58)
%DOI (SD)	18.52 (15.84)	34.81 (25.51)
Smoking (nonsmoker, former smoker, current smoker)	44/97/14	0/23/3

SD standard deviation, %DOI percent of degree of incapacity

### Data collection

A number of patients included in the study had participated in CBRP or HBRP programme more than once within the study period. These cases were identified, and only the latest available set of assessments, reflecting the collective treatment effects over the period studied, was analysed.

There are two categories of data fields. The first category consists of demographic characteristics, disease-specific information and programme participation. They include age, sex, %DOI, Body Mass Index (BMI) score, smoking history, CBRP or HBRP enrolment, number of adjunctive programmes involved, and baseline lung function (FEV_1_). The second category focuses on the outcome measures used to assess the benefits of the PR programme. They are Chronic Respiratory Questionnaire (CRQ) [27], Hospital Anxiety and Depression Scale (HADS) [28], physical (PCS) and mental (MCS) health scales of Short Form-12 (SF-12) [29], Knowledge (Additional file 2), and the 6-Min Walk Test (6MWT) [30]. As PR had not been shown to improve lung function [23–26], it was not recommended as an outcome of the PR programme according to the Quality Standards for Pulmonary Rehabilitation in Adults of British Thoracic Society [11]. Post-treatment FEV_1_ was therefore not included as an outcome for CBRP or HBRP. Baseline FEV_1_ only served as a covariate variable in this study.

### Data analyses

To ensure the relevance of the analysis, the records kept by each of the participating hospitals were pooled. Analyses of patients in the CBRP and the HBRP were conducted respectively. Paired t-tests were first used to compare the scores of the outcome measures before and after the treatment. Repeated measure analyses of covariance (rmANCOVA) were then conducted to ascertain the effects of the treatments. The patients’ baseline FEV_1_ values were the covariate. Hierarchical multiple regression analyses (stepwise) were performed to examine the relationships among the patients’ characteristics, programme participation levels, and clinical outcomes. The dependent variables were the clinical outcomes (different score between post- and pre-treatment): CRQ, HADS, SF-12, Knowledge, and 6MWT. The three blocks of independent variables were: 1) the patient’s demographic including age, gender, %DOI, smoking history, baseline FEV_1_ value, and BMI score; 2) the total number of programme participation (TOTAL) and the number of the CBRP or HBRP participation; and 3) the number of adjunctive programmes they participated in, including the CPDP, LTOT, CHCP, SMP, SMP relative, RHP, RHP relative, HLP, and HLP relative. These three blocks of variables were sequentially entered into the regression analysis. The data files used for the analyses are provided in Additional files 3 and 4.

## Results

### Differences between the pre- and post-test after CBRP and HBRP participation

Among the patients in the CBRP, pair t-tests revealed significant differences in the pre- and post-test scores on all the outcome measures except the BMI (Table 2). Significant increases in scores were found in the CRQ dyspnoea [t (154) = 4.32, *P* < 0.0001], CRQ fatigue [t (154) = 3.8, P < 0.0001], CRQ emotion [t (154) = 5.75, *P* < 0.0001], CRQ mastery [t (154) = 4.83, *P* < 0.0001] and Knowledge [t (154) = 10.61, P < 0.0001], the SF-12 PCS [t (154) = 4.39, P < 0.0001] and MCS [t (154) = 2.62, *P* = 0.01], and the 6MWT [t (154) = 12.88, P < 0.0001]. CBRP patients also showed significant decreases in HADS scores of the anxiety [t (154) = − 6.27, P < 0.0001] and depression [t (154) = − 7.42, P < 0.0001]. The results were re-examined using rmANCOVAs with the baseline FEV_1_ as the covariate.

**Table 2:** Comparisons of scores of outcome measures before and after patients participated in CBRP

Variables	Mean (SD)			*P* values		
	Before	After	95%CI	Paired t-tests	ANCOVA	Partial η^2^
CRQ Dyspnea	4.51 (1.28)	4.85 (1.33)	0.19, 0.51	< 0.0001^b^	= 0.1	0.018
CRQ Fatigue	4.41 (1.17)	4.72 (1.14)	0.15, 0.47	< 0.0001^b^	= 0.73	0.001
CRQ Emotion	5.03 (1.09)	6.45 (1.09)	0.24, 0.5	< 0.0001^b^	= 0.04^a^	0.07
CRQ Mastery	4.95 (1.4)	6.25 (1.2)	0.23, 0.55	< 0.0001^b^	= 0.04^a^	0.04
HAD Anxiety	4.75 (2.35)	2.48 (2.1)	−2.45, − 1.28	< 0.0001^b^	= 0.02^a^	0.05
HAD Depression	4.94 (4.22)	2.71 (3.39)	−2.82, − 1.63	< 0.0001^**b**^	= 0.01^a^	0.08
Knowledge	19.34 (2.9)	21.82 (2.33)	2, 2.94	< 0.0001^b^	< 0.0001^b^	0.11
SF-12 PCS	41.65 (7.95)	44.11 (8.14)	1.35, 3.56	< 0.0001^b^	= 0.047^a^	0.03
SF-12 MCS	46.4 (10.05)	48.32 (9.78)	0.47, 3.36	=0.01^a^	= 3.99	0.005
6MWT	383.61 (91.33)	443.08 (89.41)	50.35, 68.58	< 0.0001^b^	< 0.0001^b^	0.12
BMI	24.66 (18.85)	23.08 (3.38)	−4.49, 1.33	= 0.29	= 0.54	0.01

*N* = 155; 95%CI, 95% Confidence Interval of difference in means; ^a^*P* < 0.05; ^b^*P* < 0.01

Similarly, significant increases were found in scores of CRQ emotion [F (1) = 4.91, *P* = 0.04], CRQ mastery [F (1) = 4.69, P = 0.04], Knowledge [F (1) = 18.91, *P* < 0.0001], 6MWT [F (1) = 19.22, P < 0.0001,] and SF-12’s PCS [F (1) = 4.01, *P* = 0.047], and significant decreases in scores were found in the HADS anxiety [F (1) = 5.72, *P* = 0.02] and depression [F (1) = 6.41, *P* = 0.01]. However, no significant differences were revealed in the CRQ dyspnoea and the SF-12 MCS.

Among the patients in the HBRP, paired t-tests revealed significant increases in scores of Knowledge [t (25) = 3.78, P < 0.0001], SF-12 PCS [t (25) = 2.3, *P* = 0.03] and MCS [t (25) = 2.44, *P* = 0.02], and the 6MWT [t (25) = 4, P < 0.0001]. Moreover, there was a significant decrease in HAD anxiety [t (25) = − 2.95, *P* = 0.007] (Table 3). No significant changes in scores were revealed in the CRQ scales, HAD depression, and BMI. After controlled for the baseline FEV_1_, the rmANCOVA indicated significant increases only in the gain scores of Knowledge [F (1) = 6.19, P = 0.02] and the 6MWT [F (1) = 11, *P* = 0.003].

**Table 3:** Comparisons of scores on the outcome measures before and after patients participated in HBRP

Variables	Mean (SD)			*P* values		
	Before	After	95%CI	Paired t-tests	ANCOVA	Partial η^2^
CRQ Dyspnea	3.95 (1.49)	4.09 (1.48)	−0.34, 0.61	= 0.55	= 0.79	0.003
CRQ Fatigue	4.06 (1.18)	4.3 (0.93)	−0.17, 0.64	= 0.24	= 0.82	0.002
CRQ Emotion	5.13 (1.16)	5.23 (1.14)	−0.3, 0.49	= 0.63	= 0.3	0.05
CRQ Mastery	4.87 (1.25)	5.01 (1.24)	−0.25, 0.53	= 0.46	= 0.74	0.005
HAD Anxiety	4.38 (4.02)	2.81 (3.74)	−2.68, −0.47	= 0.007^b^	= 0.31	0.042
HAD Depression	4.15 (4.67)	4.27 (5.17)	−1.07, 1.3	= 0.84	= 0.57	0.014
Knowledge	20.08 (2.47)	21.92 (1.57)	0.84, 2.85	= 0.001^b^	= 0.02^a^	0.21
SF-12 PCS	40.8 (7.24)	44.01 (8.48)	0.34, 6.08	= 0.03^a^	= 0.93	0.0001
SF-12 MCS	43.18 (10.9)	47.85 (9.57)	0.73, 8.62	= 0.02^a^	= 0.37	0.03
6MWT	256.27 (110.37)	303.88 (125.98)	23.08, 72.15	< 0.0001^b^	= 0.003^b^	0.31
BMI	22.16 (3.64)	22.08 (3.4)	−0.32, 0.17	= 0.54	= 0.7	0.01

*N* = 26; 95%CI, 95% Confidence Interval of difference in means; ^a^*P* < 0.05; ^b^*P* < 0.01

### Factors influencing the outcomes of pulmonary rehabilitation Programmes

**Fig. 1:**
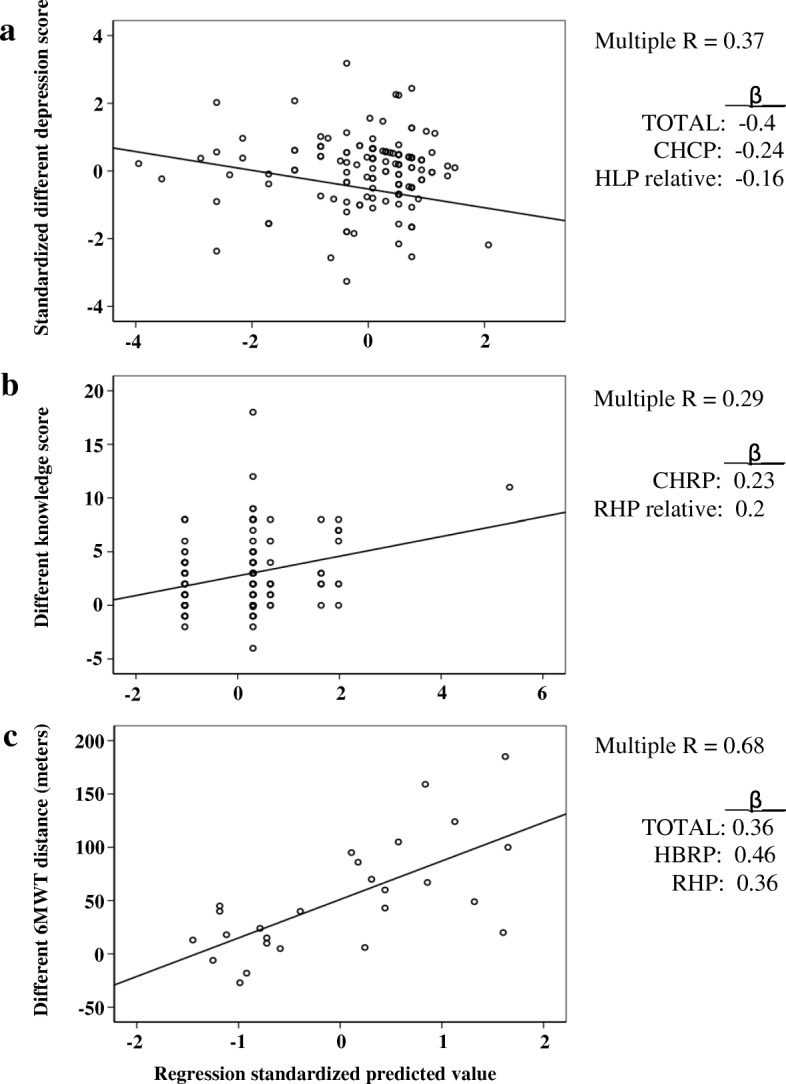
Scatterplots illustrating the relationships between outcome variables and program participations. **a** The y-axis represents the standardized different HAD depression scores in CBRP patients and the x-axis represents the standardized predicted values of program participations including TOTAL, CHCP, and HLP relative. **b** The y-axis represents different knowledge scores in CBRP patients and the x-axis represents standardized predicted values of CBRP and RHP relative program participations. **c** The y-axis represents the different distance of 6MWT in meters in HBRP patients and the x-axis represents the standardized predicted values of program participations including TOTAL, HBRP, and RHP. Multiple R, correlation coefficient for multiple regression; β, standardized coefficient of each independent variable

For patients in the CBRP, the different scores in HAD depression were predicted by the TOTAL, CHCP, and HLP relative (Multiple *R* = 0.37, R^2^ = 0.14). Based on this model, a 1.0-episode increase in the number of family members participating in the HLP was associated with a 1.76-point decrease in the HAD depression score; a 1.0-unit increase in patients’ CHCP participation was associated with a 1.15-point decrease in HAD depression score; and a 1.0-unit increase in TOTAL was associated with a 0.31-point decrease in HAD depression score (Table 4A and Fig. 1a). The different scores in Knowledge were predicted by the number of participations in CBRP and RHP relative (Multiple *R* = 0.29, R^2^ = 0.09). The model illustrated that a 1.0-episode increase in the RHP relative was associated with a 1.54-point increase in the Knowledge score; and a 1.0-unit increase in the CBRP participation was associated with a 1.23-point increase in the Knowledge score (Table 4b and Fig. 1b). No other significant regression model was found.

**Table 4:** Results of hierarchical multiple regression analyses

	CBRP	(*N* = 155)					
	Predictor	B	β	R	R^2^	ΔR^2^	ΔF
A.							
	DV: Different score in HAD depression						
Model 1	TOTAL	−0.21	− 0.28	0.28	0.08	0.08	12.63^b^
Model 2	TOTAL	−0.28	−0.37	0.34	0.12	0.04	6.97^b^
	CHCP	−1.05	−0.22				
Model 3	TOTAL	−0.31	− 0.4	0.37	0.14	0.02	4.02^a^
	CHCP	−1.15	−0.24				
	HLP relative	−1.76	−0.16				
B.							
	DV: Different score in knowledge						
Model 1	CBRP	1.14	0.21	0.21	0.04	0.05	7.08^b^
Model 2	CBRP	1.23	0.23	0.29	0.09	0.04	6.77^a^
	RHP relative	1.54	0.2				
	HBRP	(*N* = 26)					
C.							
	DV: Different score in 6MWT						
Model 1	TOTAL	2.77	0.41	0.41	0.17	0.17	4.92^a^
Model 2	TOTAL	3.05	0.45	0.59	0.35	0.18	6.07^b^
	HBRP	38.53	0.42				
Model 3	TOTAL	2.38	0.36	0.68	0.47	0.12	6.39^**^
	HBRP	41.96	0.46				
	RHP	52.23	0.36				

DV, dependent variable; B, unstandardized coefficient; β, standardized coefficient

^a^*P* < 0.05; ^b^*P* < 0.01

For patients in the HBRP, the 6MWT was the only outcome variable that was predicted by the TOTAL and the number of participation in HBRP and RHP (Multiple *R* = 0.68, R^2^ = 0.47). Based on this model, a 1.0-unit increase in the RHP participation was associated with a 52.23-m increase in the 6MWT. This is followed by a 1.0-unit increase in the HBRP participation, which was associated with a 41.96- m increase in the 6MWT. Furthermore, a 1.0-unit increase in TOTAL was associated with a 2.38-m increase in the 6MWT (Table 4c and Fig. 1c).

## Discussion

To our knowledge, this study is the first to report the outcomes of community-based and home-based pulmonary rehabilitation programmes provided by the same teams of rehabilitation professionals from three separate hospitals. Therefore, the treatment outcomes of the two types of programmes are very comparable. This reveals specific strengths and weaknesses associated with each programme. This is also the first report of pulmonary rehabilitation treatment outcomes independent of patients’ baseline lung functions, particularly regarding the importance of patients’ participation in influencing the physical and psycho-social aspects of the treatment outcomes on pneumoconiosis patients.

The results suggested that the CBRP had positive effects in enhancing the patients’ HRQOL (CRQ fatigue, emotion, and mastery) and reducing their psychological symptoms (HADS anxiety and depression). Patients who participated in the CBRP were found to show improvement in their knowledge about the disease as well as the exercise capacity (6MWT). The findings on the improvement in the patients’ quality of life are consistent with those of previous studies [13, 31]. However, the finding that the CBRP did not improve the CRQ dyspnoea score is inconsistent with findings reported in previous studies [32, 33]. A plausible reason for this discrepancy is that this study incorporated patients’ baseline lung function as a covariate, which was not the case in previous studies. Future studies should further explore how a patient’s lung function, particularly different levels of initial lung capacities, would influence the treatment outcomes of PR programmes.

Several studies proposed that social support [31, 34–36] embedded in community-based PR programmes contributes towards the improvement in patient’s psychological symptoms [31, 36, 37]. The results of this study further substantiate this proposition. A higher number of home visits (CHCP) made to the patients, as well as their relatives having attended educational talks more frequently (in HLP) were factors found to be significantly associated with the reduction of depression symptoms among patients who completed the CBRP. The CHCP consisted of home visits by healthcare professionals to monitor the health and psycho-social statuses of patients (see content in Additional file 1). The HLP involved educational talks to patients and their relatives on self-maintenance and healthy lifestyles. The RHP provided lectures on pneumoconiosis and respiratory hygiene. Open to both patients and their relatives, the talks were arranged by NGOs but conducted by healthcare professionals. These classes were useful for enhancing patient’s knowledge on the disease. This postulation is supported by findings on the significant relationships among the relatives’ participation in the CBRP and RHP and patient’s gain in the knowledge (Fig. 1b).

Apart from learning about the disease, the patients showed improvements in mobility function after participating in the CBRP. A mean improvement of 59.5 m in 6MWT was found to exceed the clinical threshold of 54 m set in other studies [38, 39]. Our findings on mobility, as general exercise capability, are consistent with those reported in other studies on community-based programmes, which considered mobility as an important outcome to patients with pneumoconiosis [13, 33].

In general, the effects of the HBRP were more modest than those of the CBRP. After controlling for patients’ baseline lung capacities, significant improvements were found in patients’ knowledge about the disease and in exercise capacity after completing the HBRP. The improvements in exercise capacity after completing the HBRP were consistent with previous findings [14, 15, 17, 40]. Patients who completed the HBRP showed a mean increase of 47.6 m on the 6MWT, which is below the clinical threshold of improvements suggested in other studies [38, 39]. This is perhaps because patients in the HBRP were of older age, had greater baseline %DOIs, and lower lung capacities than those in the CBRP. Of note, the HBRP did not appear to produce significant positive effects in improving patients’ health-related quality of life and psychological symptoms. These findings are inconsistent with those reported in previous studies on home-based programmes [14, 15, 40, 41]. This inconsistency could have been due to the small sample size of the HBRP group. Previous studies indicated that patients of home-based programmes valued social support from and interactions with professionals, families, and peers [42–44]. A recent study suggested that home-based programmes should aim at improving physical capacities in order for patients to progress and participate in community-based programmes, which bring stronger psychosocial benefits [17].

The improvement of the 6MWT in HBRP patients was related to the total number of programmes of HBRP and RHP that the patients had participated in (Table 4c and Fig. 1c). Pulmonary rehabilitation consists of many programmes that help improve patients’ physical functions (Additional file 1). It is likely for patients who had participated in more PR programmes to gain more benefits, thus performing better in the 6MWT. The HBRP involved physical and respiratory training, which improved patients’ exercise tolerance levels [14]. A longer training period was found to be more effective in enhancing the physical functions [31]. In the RHP, therapists taught patients about pneumoconiosis, respiratory hygiene, the use of inhalers, and energy conservation. These resulted in better health management [45] and the ability to achieve greater exercise tolerance levels for those who participated more frequently.

### Limitations

The data obtained for this study was based on convenient sampling, hence, the findings should be interpreted with caution. Generalization of results to other groups of patients with pneumoconiosis would therefore be limited. The study was not a randomized controlled trial. Thus, the treatment effects reported showed, at best, trends in improvements.

No information on the medications taken by the patients was included in the data. Nevertheless, the common practices of all the case medical officers who referred the patients for enrolment in the community- or home-based rehabilitation programmes were: 1) patient was referred when the medications were deemed optimized for the symptom control; and 2) the medications typically prescribed to the patients included various types of inhaled bronchodilators. Despite taking a relatively unified approach to the medication prescriptions, the possibility that the differences in the outcomes among the patients between the two programmes due to the differences in the medications taken by the patients cannot be completely excluded.

Another drawback is that the patients’ data was under-reported. This is rather common in studying outcomes of pulmonary rehabilitation among patients with COPD [14, 46]. The 181 completed cases out of the 685 total cases may not fully represent the typical patients receiving the services. Many of the patients had repeatedly participated in the CBRP or HBRP, so the treatment effects could have been inflated. Further studies should generate evidence on the efficacy of these programmes by employing a more stringent research design and larger sample size.

## Conclusion

Patients with pneumoconiosis require long-term rehabilitation services. To best fulfil their needs, rehabilitation programmes are offered in the community or at home. The patients were found to show positive gains in areas of knowledge, exercise tolerance, quality of life, and psychological symptoms after attending community-based programmes. Some of these gains were related to patients’ attendance frequency levels of adjunctive programs and the involvement levels of their relatives in the treatment processes. Home-based programmes, in contrast, produced less obvious treatment effects, particularly regarding quality of life and psychological symptoms. The findings suggest the importance of strengthening psycho-social intervention for patients who take part in home-based rehabilitation programmes.

## Additional files

Additional file 1: Description of Pulmonary Rehabilitation (PR) Services for Patients with Pneumoconiosis in Hong Kong. (DOCX 26 kb)

Additional File 2: Description of the Knowledge test. (DOCX 16 kb)

Additional file 3: File name: CBRP.xlsx. Title of data: Community based rehabilitation program. The file contains the data of treatment outcomes for patients who participated in community based pulmonary rehabilitation program. (XLSX 59 kb)

Additional file 4: File name: HBRP.xlsx. Home based rehabilitation program. The file contains the data of treatment outcomes for patients who participated in home based pulmonary rehabilitation program. (XLSX 16 kb)

## Abbreviations

%DOI: Percentage of the degree of impairment
6MWT: 6-Min Walk Test
BMI: Body Mass Index
CBRP: Community-based rehabilitation programmes with maintenance pulmonary rehabilitation programmes
CHCP: Comprehensive Home Care Program
CPDP: Comprehensive Post Discharge Program
CRQ: Chronic Respiratory Questionnaire
FEV_1_: Baseline forced-expiratory volume
HADS: Hospital Anxiety and Depression Scale
HBRP: Home-Based Rehabilitation Program
HLP relative: HLP participated by patients’ relatives
HLP: Healthy Lifestyle Program
HRQOL: Health-related quality of life
LTOT: Long Term Oxygen Therapy
MRP: Maintenance Rehabilitation Program
NGO: Nongovernmental organization
PR: Pulmonary rehabilitation
RHP relative: RHP participated by patients’ relatives
RHP: Respiratory Hygiene Program
SF-12’s PCS and MCS: Health survey Short Form-12’s physical and mental components
SMP relative: SMP participated in by the patients’ relatives
SMP: Self-Management Program
TOTAL: Total number of programme participation (core plus adjunctive programmes)
