# Supplementary material for: Outcomes of community-based and home-based pulmonary rehabilitation for pneumoconiosis patients: a retrospective study
Source: BMC Pulm Med. 2018 Aug 9;18:133. doi: 10.1186/s12890-018-0692-7 (PMC6085700; doi:10.1186/s12890-018-0692-7)
Supplement: Supplementary file 1 — Description of Pulmonary Rehabilitation (PR) Services for Patients with Pneumoconiosis in Hong Kong. (DOCX 26 kb) [file 12890_2018_692_MOESM1_ESM.docx]

**Additional file 1:** Description of Pulmonary Rehabilitation (PR) Services for Patients with Pneumoconiosis in Hong Kong

There are two core PR programmes, including Community Based Rehabilitation Programme (CBRP) and Home Based Rehabilitation Programme (HBRP). Maintenance Rehabilitation Programme (MRP) consists of ‘refresher classes’ organised for those that have completed the CBRP for at least 6 months. Adjunctive programmes that serve the specific needs of patients and provide education for patients include Long Term Oxygen Therapy (LTOT), Comprehensive Post Discharge Program (CPDP), Comprehensive Home Care Program (CHCP), Pulmonary Rehab Program (PRP), Healthy Lifestyle Program (HLP), Self-Management Program (SMP), and Respiratory Hygiene Program (RHP).

| Names of program | Description of services |
| --- | --- |
| CBRP | These programmes consist of training classes that promote patients’ physical and mental well-being comprehensively. They are arranged in community centres near the patients’ home. The programme is 4to 6 weeks in length with classes on a biweekly basis. Each class is t2 hours long and is targeted at 5 patients.  Physiotherapists are responsible for delivering 8 hours of services, involving breathing retraining*, bronchial hygiene^@^, dyspnea management^#^, home exercise conditioning^$^, assessment of oxygen requirement on exertion by oximetry, and other services including pain management, walking aids and exercise apparatus prescription as indicated by patients.  Occupational therapists are responsible for 3 hours of services involving health education on topics including basic principles of energy conservation techniques (ECT)^%^ and coordinated breathing (CB)^ in self-care, application of ECT and CB in household chores and community living skills, panic control skills^¥^, stress management and relaxation techniques^£^.  There are 3 hours of nursing services, involving health education topics, includingpneumoconiosis and complication, drug compliance, puff technique, smoking cessation, nutrition, prevention of chest infection, dyspnea management, and vaccination. The remaining hours are devoted to social worker services (2 hours) and outcome assessments (pre- and post-treatment, 4 hours). |
| HBRP | This is to serve patients with physical conditions too poor to join community-based programme. This programme mainly provides physical and mental support to homebound patients and all outcome assessments are optional. This programme is composed of a total of 8 home visits. Each visit lasts for 1 to 1.5 hours.  Physiotherapists are responsible for 4 home visits where services involve breathing retraining*, bronchial hygiene^@^, dyspnea management^#^, home exercise conditioning^$^, assessment of oxygen requirement on exertion by oximetry, and other services including pain management, walking aids and exercise apparatus prescription as indicated by the patient.  Occupational therapists are responsible for 2 home visits, providing training and services on home modification^€^, monitor activities of daily living assessment with ECT^%^ and CB^, aid/ adaptive device prescription recommendation^®^, assessment of appropriate and safely use of LTOT equipment, carer training^±^, relaxation training^£^, coping skills in panic control^¥^, on-site community living skills assessment and training^©^, LTOT assessment and recommendation, and recommendation or prescription of wheelchair.  Nurses are responsible for 2 home visits, involving services related to health status, medical and medication reviews.  The remaining 2 home visits are devoted to social worker services. |
| MRP | This is a refresher programme for patients that havecompleted CBRP for at least 6 months. Like the CBRP, MRP classes are arranged in community centres near the patients’ home. MRP is 2 weeks in length and consists of 6 classes. Each class is 2 hours long and is composed of 10 patients. MRP has the same service contents as the CBRP. |
| CPDP | This is too serve patients recently discharged from hospitals. The overall aim is to decrease hospital readmission rate within 28 days after discharge. CPDP is composed of a total of 6 home visits. Each visit takes 1 to 1.5 hours.  A nurse performs the first home visit within a week after discharge to perform health status examination, medical and medication reviews.  A physiotherapist performs home visits once a week for 2 weeks and teaches breathing retraining* and conditioning exercise^$^.  An occupational therapist performs home visits once a week for 2 weeks and provides advice on home modification^€^, monitor activities of daily living assessment with ECT^%^ and CB^, aid/ adaptive device^®^ prescription recommendation, assessment of appropriate and safely use of LTOT equipment, carer training^±^, relaxation training^£^, and coping skills in panic control^¥^.  A social worker performs 1 home visit. |
| LTOT | This is for patients with severe dyspnea and difficulty in breathing, LTOT refers to the provision of oxygen therapy for continuous use at home. It involves the use of nasal cannula to deliver a continuous flow of oxygen to the patient. |
| CHCP | At least 2home visits would be paid to obtain updated information regarding partients’- health and psychosocial status, and to recommend or implement intervention or prevention programmes as appropriate. Each visit takes approximately 1.5 hours. |
| SMP | This is one-to-one health consultation provided by medical staff to provide patients with self-management plans on managing possible exacerbation of underlying lung disease and minimising hospital admission. SMP is provided to patients that have joined CPDP, HBRP, LTOT, CHCP, CBRP or MRP. CBRP patients typically receives 1 SMP session, whereas other patients typically receive more than 1 SMP session. Each session takes approximately 1.5 hours. |
| RHP | This is a class on general knowledge on pneumoconiosis and respiratory hygiene taught by a nurse from an NGO. During the class, a pamphlet would be distributed to each patient, explaining the techniques of pneumoconiosis management. This includes respiratory hygiene, use of drugs and inhalers, and energy conservation methods. The pamphlet includes a telephone hotline for enquiries on social, rehabilitation, and medical problems. Relatives of the patients are welcome to attend the RHP along with the patients. RHP is a session of atalk that takes approximately 45 minutes. |
| HLP | These are talks on health education relating to various relevant topics. The talk is held in a community centre and takes approximately 45 minutes. |

*Breathing retraining involves exercises, including relaxed breathing, purse lip breathing, diaphragmatic breathing, relaxed positioning, relaxation and coordinated breathing.

^@^Bronchial hygiene involves the training in exercises, such as huffing, coughing, or the Active Cycle of Breathing Techniques. Exercise training can be tailor-made and adjusted to the patients depending on their assessment and continuous health reviews.

^#^Dyspnea management involves the use of ventilation and movement strategies to facilitate breathing, including active assisted cough techniques, volume augmentation such as breath stacking, body position, and ventilation patterns.

^$^Conditioning exercise is exercise and practice to rebuild the body after discharge. The exercises can be customized to serve the specific needs of patients depending on continunous assessment and health reviews. For HBRP or CPDP patients that are less mobile or homebound, the exercises used range from limb mobilizing exercises while sitting, stepping exercise with upper limb support to walking. For those patients that are more mobile, the exercises used range from walking, Tai Chi, muscles strengthening, to aerobic exercises.

^%^Energy conservation techniques involve education and training on organizing daily schedule, simplifying tasks, using proper postures to conserve energy when performing daily activities.

^Coordinated breathing involves education and training on the practice of breathing techniques, such as pursed-lip breathing and diaphragmatic breathing to reduce fatigue by regulating the air into and out of the lungs during the performances of strenuous activities.

^¥^Coping skills in panic control involves education and training on using adaptive methods to meet the demands of daily activities, prevent and manage panic attacks.

^£^Relaxation training involves the education and training of topics, including internal locus of control, stress and anxiety release essential for the self-management of pneumoconiosis.

^€^Home modification involves reorganization of living spaces to increase usage, safety, security, and independence for helping the patients to adapt to their limitations imposed by pneumoconiosis.

^®^Aids/ adaptive devices involve the evaluation of the need, identification, provision, fitting, customization, and training on the use and care for the devices, such as wheelchair, bathboard, bedside commode seat, handrail, and/ or long-handled reacher etc., to facilitate everyday activities.

^±^Carer training involves education and training of skills in taking care of the patient, particularly in the management of patient’s dyspnea and fatigue associated with pneumoconiosis.

^©^On-site community living skills assessment and training involves conducting on-site (patients’ home or neighborhood in the community) visits to assess and improve the patients living skills fundamental to the patients’ functioning at home or in the community. Living skills at home includes doing completing household chores, such as cooking, cleaning, and washing dishes etc. whereas in the community involves going to the supermarket, shopping centre, parks or community centre, and taking the public transport etc.
