# Supplementary material for: Outcomes of community-based and home-based pulmonary rehabilitation for pneumoconiosis patients: a retrospective study
Source: BMC Pulm Med. 2018 Aug 9;18:133. doi: 10.1186/s12890-018-0692-7 (PMC6085700; doi:10.1186/s12890-018-0692-7)
Supplement: Supplementary file 2 — Description of the Knowledge test. (DOCX 16 kb) [file 12890_2018_692_MOESM2_ESM.docx]

**Additional File 2:** Description of the Knowledge test

The Knowledge test (Knowledge) is a custom-designed instrument of measuring the health knowledge of patients with pneumoconiosis. There are 25 items covering topics on symptom management, breathing technique, and energy conservation strategies during exercises. The responses are true or false. The patients were to give the most appropriate responses (a check on the answer sheet) based on their knowledge gained from the CBRP/MRP or HBRP. The maximum score is 25. The higher the score indicates better knowledge about pneumoconiosis and its management.
